# Continuous microfluidic assortment of interactive ligands (CMAIL)

**DOI:** 10.1038/srep32454

**Published:** 2016-08-31

**Authors:** Yi-Hsing Hsiao, Chao-Yang Huang, Chih-Yung Hu, Yen-Yu Wu, Chung-Hsiun Wu, Chia-Hsien Hsu, Chihchen Chen

**Affiliations:** 1 Institute of Nanoengineering and Microsystems, National Tsing Hua University, Hsinchu 30013, Taiwan; 2 Institute of Biomedical Engineering and Nanomedicine, National Health Research Institutes, Miaoli 35053, Taiwan; 3 Development Center for Biotechnology, New Taipei City 22180, Taiwan; 4 Department of Power Mechanical Engineering, National Tsing Hua University, Hsinchu 30013, Taiwan

## Abstract

Finding an interactive ligand-receptor pair is crucial to many applications, including the development of monoclonal antibodies. Biopanning, a commonly used technique for affinity screening, involves a series of washing steps and is lengthy and tedious. Here we present an approach termed continuous microfluidic assortment of interactive ligands, or CMAIL, for the screening and sorting of antigen-binding single-chain variable antibody fragments (scFv) displayed on bacteriophages (phages). Phages carrying native negative charges on their coat proteins were electrophoresed through a hydrogel matrix functionalized with target antigens under two alternating orthogonal electric fields. During the weak horizontal electric field phase, phages were differentially swept laterally depending on their affinity for the antigen, and all phages were electrophoresed down to be collected during the strong vertical electric field phase. Phages of different affinity were spatially separated, allowing the continuous operation. More than 10^5^ CFU (colony forming unit) antigen-interacting phages were isolated with ~100% specificity from a phage library containing 3 × 10^9^ individual members within 40 minutes of sorting using CMAIL. CMAIL is rapid, sensitive, specific, and does not employ washing, elution or magnetic beads. In conclusion, we have developed an efficient and cost-effective method for isolating and sorting affinity reagents involving phage display.

Characterization of interactions between molecule pairs (e.g., ligands to receptors, antibodies to antigens, enzymes to substrates, and aptamers or drugs to their targets, etc.) provides the key to a wide spectrum of applications, including drug development, genetic engineering, biochemistry, biotechnology, molecular biology, etc.^1^,^2^,^3^,^4^,^5^,^6^. Phage display, in which the foreign amino acid sequence is genetically fused to the viral coat protein and displayed on the outer surface of the bacteriophage, provides a physical linkage of diverse phenotypes with their individual genotypes. Large libraries of proteins or peptides can be screened against target molecules, such as antigens or nucleic acids in a process analogous to natural selection. Phage display has been instrumental for conducting *in* *vitro* evolution and the development of targeted therapies, such as engineered monoclonal antibodies^7^,^8^,^9^.

However, the selection of phage libraries against target antigens remains a challenge, and is most commonly achieved using the biopanning technique. Biopanning involves the immobilization of target molecules or antigens onto a solid support or magnetic beads followed by the incubation with the phage library. After extensive washing to remove nonadherent or weakly binding phages, bound phages are eluted and recovered using elution buffer, and may be subsequently amplified in a bacteria host for further enrichment^10^,^11^,^12^. Three to five repeated rounds of incubation, washing, elution, and amplification steps are usually carried out in order to obtain high-affinity phages, which can be quite laborious, lengthy and experimentally challenging for a number of reasons. First, non-specific phages are recovered when phage libraries are incubated with immobilized antigens. Second, removal of background by repeated washes is both labor-intensive and inefficient. Third, antigens and potential ligands can be lost during the washing steps required.

We reason that the sorting efficiency can be improved if the stochastic encounters between phages and antigens in the discrete steps of biopanning can occur in a much more frequent and controlled manner. Microfluidic technology confers advantages, including the precisely controlled fluidic environment and amiability to be automated, and has been developed and applied for molecular separations^13^,^14^,^15^ and detections^16^,^17^,^18^. For example, it has been shown that microfluidic devices can provide a much more accurate and automatic handling of fluids in the biopanning procedure^19^,^20^,^21^,^22^,^23^,^24^. However, multiple rounds of biopanning are still needed to obtain desired performance and/or throughput. We have devised a method termed continuous microfluidic assortment of interactive ligands (CMAIL), exploiting the fact that most phage display work has used filamentous phage strains such as F1, Fd and M13 bacteriophages, which are closely related, small, of unique elongated shape, and inherently negatively charged since the carboxyl-terminal of the major coat protein pVIII interacts with the virion DNA and is shielded from the solvent^25^,^26^,^27^. CMAIL allows continuous separation of antigen-interactive phages from remaining noninteractive phages; this is accomplished by differential electrophoresis under two alternating orthogonal electric fields that drive phages across the hydrogel matrix coated with antigens. A weaker electric field pulls noninteractive phages laterally, while a stronger electric field drives phages downstream to be collected at different locations as schematically shown in Fig. 1a.

Alternately pulsed electric fields at an angle have been utilized to perform the size-based fractionation of DNA molecules in gels^28^ or an array of micropillars^29^. Larger DNA molecules have to reorient themselves in response to alternating electric fields and hence migrate less efficiently than smaller ones. In CMAIL, phages are of the same size, but are spatially separated based on their affinity for the target antigen. CMAIL, eliminating the need for repeated washing steps, can operate continuously. In addition, no elution of bound phages from a surface or a magnetic bead is required. Here we report in detail the performance of CMAIL using prescreened phage clones and the phage library. Our data compare favorably to results of biopanning using magnetic beads, and suggest that selection antigen-interactive scFv using CMAIL may enable rapid identification of promising antibodies for targeted therapies among other applications.

## Results

### Immobilization of antigen molecules to the agarose gel is stable under the electric field

Agarose gels were functionalized with target antigen molecules for the sorting of interactive phages. The immobilization of antigens was achieved using hetero-crosslinking molecules whose ends were photo-reactive and amine-reactive, respectively. The conjugation of antigen molecules to the agarose gel was assessed using fluorescence-based readouts. We applied fluorescently labelled antibodies against the antigen to the functionalized agarose gel and operated the CMAIL device at an electric field of 7.4 V/cm, which was the highest intensity of electric field used in this study. As shown in Supplementary Figure 1, the fluorescence intensity remained stable for at least 120 minutes evaluated, suggesting stable immobilization of antigen molecules even under the electric filed. The initial drop in fluorescence intensity may be due to the removal of unbound antibodies by the electric field since there was no washing step conducted.

### A stronger electric field is needed to electrophorese phages of higher affinity for the antigen

Filamentous phages have been shown to migrate through 2% agarose gels at pH 9.45, and the electrophoretic mobility is dependent mainly on the net charge of the pVIII coat protein for phages of the same size^27^,^30^. We examined the electrophoretic migration of M13 phages expressing fusion proteins through the 1% agarose gel at physiological pH, pH 7.4. Fluorescently labelled phage clone M+ (labelled green, antigen-interactive) and M− phages (labelled red, antigen-noninteractive) were loaded into the 1% agarose gel coated with target antigens. Fluorescent micrographs were utilized to visualize phages and suggested that both M+ and M− phages migrated toward the anode. An electric field of 3.6 V/cm or less was enough to drive M− phages, while 6.0 V/cm could drive M+ phages as shown in Supplementary Figure 2.

### Phage numbers may be evaluated using nanoparticle tracking analysis (NTA)

Enumeration of phages has been demonstrated using the established technology of nanoparticle tracking analysis (NTA)^31^, which is more rapid than conventional phage titer analyses. NTA is an image-based method for sizing and concentration measurement of nanoscale particles. NTA provides the total count of particles under the scattering mode, and has the capability to analyze fluorescently labeled particles under the fluorescence mode. We first compared phage counts obtained by using NTA and conventional phage titer analyses. Results, summarized in Supplementary Figure 3a, were in good agreement with each other for phage samples of concentrations ranging from 10^7^ to 10^11^ CFU/mL. However, when the concentration of phages was below 10^7 ^CFU/mL, the number of particles per field of view was too low to be determined reliably using NTA. Next, we evaluated the concentration of fluorescently labeled phages. Phage counts obtained under the scattering mode and fluorescence mode of NTA were statistically insignificant (Supplementary Figure 3b), which validated the settings of NTA for counting fluorescently labeled phages. We then mixed together fluorescently-labeled M+ phage and unlabeled M− phage samples of known concentrations. The concentration of total phages was determined under the scattering mode, while fluorescently labelled phages were selectively identified under the fluorescence mode. The unlabeled phage count was subsequently calculated using the difference of the total phage count and the fluorescently labeled phage count, and was comparable to the concentration of unlabeled M− phages measured before mixing (Supplementary Figure 3c). These results provide validation of NTA as a technique for counting both unlabeled and fluorescently-labeled phage subpopulations and was employed for assessing the performance of CMAIL.

### Phage clones are sorted using CMAIL

**Figure 1:**
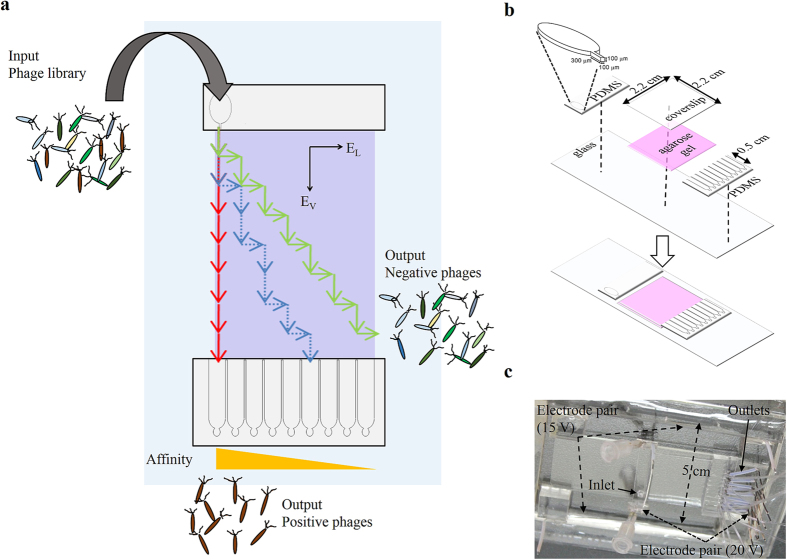
Operation and fabrication of the CMAIL device. (**a**) Schematic rendition of the device; phages are loaded from the inlet and electrophoresed inside the agarose gel coated with target antigens under two alternating electric fields, *E*_*L*_ and *E*_*V.*_ *E*_*V*_ is greater than *E*_*L*_. Negative, or antigen-noninteractive, phages migrate diagonally across the gel under both electric fields, while positive, or antigen-interactive, phages migrate only when the stronger electric field *E*_*V*_ is applied. Phages are sorted and collected at different outlets continuously based on their affinity for the target antigen. (**b**) The PDMS inlet and outlets are replicated from a master mold made by using conventional lithography, and are chemically bonded to a glass slide. Agarose gel is dispensed and cured in the center reservoir. (**c**) Image of the CMAIL device.

**Figure 2:**
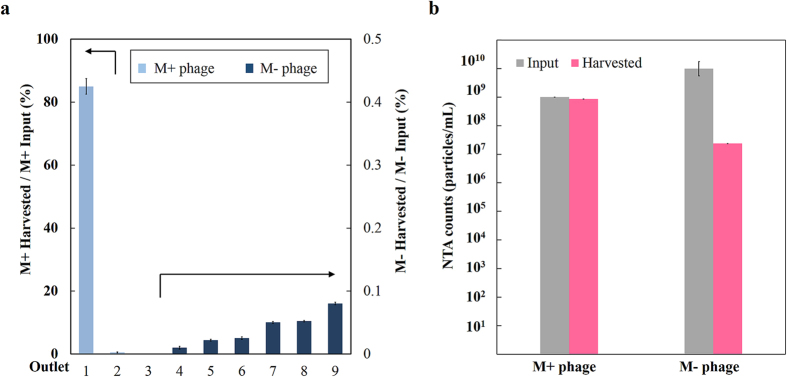
Antigen interactive (M+) and noninteractive (M−) phages were sorted into different outlets using CMAIL. The input sample contained M+ and M− phages at concentrations of 10^9^ and 10^10^ CFU/mL, respectively. (**a**) Most of the input M+ phages were recovered at outlet 1, and very few M− phages were collected at outlets 4~9. Phage counts of samples at outlets 2 and 3 were too low to be determined reliably using NTA. (**b**) Phage counts by using NTA indicated that more than 85% of input M+ phages were recovered, while less than 0.24% of input M− phages were collected at all the outlets combined.

The schematics and fabrication of the CMAIL device were shown in Fig. 1a,b, respectively. An image of the completed device was shown in Fig. 1c. We set out to study antigen binding by using CMAIL to test an established antigen-antibody fragment pair. Experiments were designed using cloned M+ and M− phages (displaying the antibody fragment selected to have, respectively, a high and low affinity for the antigen (vascular endothelial growth factor receptor 2 (VEGF-R2)). We reasoned that antigen-interactive M+ phage could interact with antigens coated in the gel strongly and migrate little under the weak horizontal electric filed, *E*_*L*_. Consequently, M+ phages would be recovered at outlet 1, which was directly downstream from the inlet. In contrast, antigen-noninteractive M− phages would move diagonally across the hydrogel under both electric field phases. Since phage virions collected at outlets were to be enumerated using NTA, M+ phages were fluorescently labeled, and then mixed with M− phages at 1:10 ratio, or 10^9^:10^10^ CFU/CFU. The sample mixture was introduced into a CMAIL device, in which two orthogonal electric fields, *E*_*L*_ and *E*_*V*_, alternating every minute for 40 minutes were applied across the gel. *E*_*V*_ was greater than *E*_*L*_ and along the direction from inlet to outlet channels. As shown in Fig. 2a, outlet 1 collected the most phages and nearly all of them were M+ phages. Only a few M− phages were counted at outlets 4~9. A total of 8.5 × 10^8^ CFU M+ phages were harvested from the outlets, which was equivalent to a recovery rate of 85%. In contrast, only 0.2%, or 2 × 10^7^ CFU M− phages were collected at all outlets (Fig. 2b). To further confirm phage counts obtained by using NTA, conventional phage titer analyses and DNA sequencing were conducted. Recovered phages were isolated as described in Methods. The DNA sequences of randomly selected bacteria colonies were determined for 40 isolates; 10 each were infected from phages recovered in four outlets 1, 2, 3 and 6, respectively (Supplementary Figure 4). DNA sequence data, summarized in Table 1 and Supplementary Table 1, showed that all of the sequenced colonies infected with phages recovered at outlet 1 contained the sequence of M+ phages, while only 8 out 9 successfully sequenced colonies were positive for M+ phages from outlets 2, 3 and 6.

### More than 80% antigen-interactive phages are recovered when the occurrence is above 1/10,000

**Figure 3:**
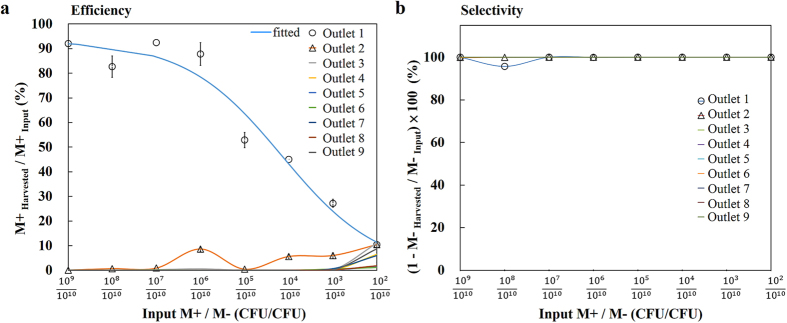
Sorting of antigen interactive (M+) and noninteractive (M−) phages using CMAIL is efficient and specific. The input sample contained M+ phages ranging from 10^2^ to 10^9^ CFU and M− phages at a constant number of 10^10^ CFU. Hence, the mixing ratio of input M+ to M− phages ranged from 10^−8^ to 10^−1^ accordingly. Phages collected at outlets 1 to 9 were counted separately using conventional phage titer analyses (Supplementary Figure 5a). ELISA analysis was used to assess the percentage of bacterial colonies infected by M+ phages (Supplementary Figure 5b). (**a**) Most M+ phages were recovered at outlet 1 for the various mixing ratios of input M+ to M− phages evaluated. The efficiency, or the percentage of recovered M+ phages, remained greater than 80% when the mixing ratio was above 10^−4^, and dropped gradually to 10% when the mixing ratio was 10^−8^. (**b**) The specificity, or 100% minus the percentage of recovered M− phages, remained close to 100% for samples at all the outlets and mixing ratios evaluated.

Antigen-interactive M+ phages were mixed with antigen-noninteractive M− phages at various ratios ranging from 1:10 to 1:10^8^ to further assess the performance of CMAIL. Sorted phages were collected at each outlet and were counted separately using conventional phage titer techniques. Bacterial colonies infected by M+ phages were further confirmed using ELISA (see Method and Supplementary Figure 5b). As indicated in Fig. 3a and Supplementary Figure 5a, most phages were recovered at outlet 1, the outlet that was in-line with the inlet. Only a small fraction of phages were collected at other outlets. The efficiency, or the percentage of recovered M+ phages to input M+ phage remained to be greater than 80%, even when the ratio of input M+ to M− phage was 1:10^4^. The efficiency decreased gradually with the lower mixing ratios, and dropped to about 10% when the number of input M+ phages was 10^2^ CFU. The selectivity, defined as 100% minus the percentage of harvested M− phages to input M− phages, was used to assess the capability of CMAIL to exclude antigen-noninteractive phages. As indicated in Fig. 3b, the selectivity was close to 100% for all the mixing ratios evaluated.

### Phages were enriched at different outlets based on their affinity

**Figure 4:**
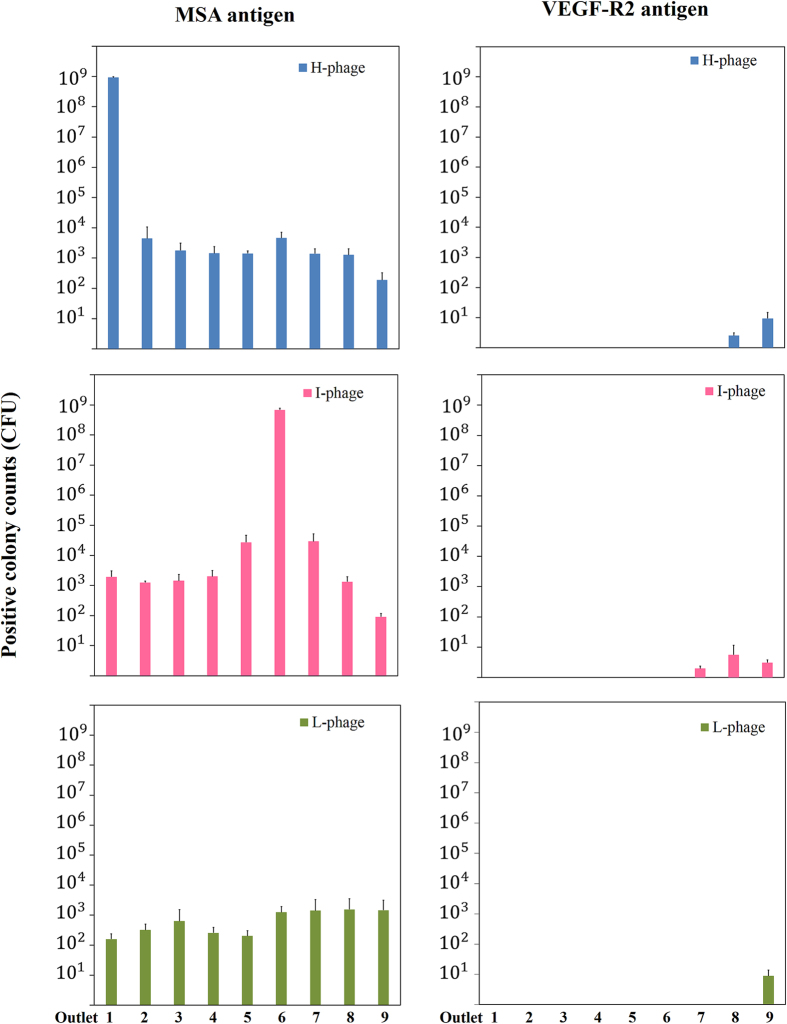
Phage clones of different affinity for the antigen are spatially separated in the CMAIL device. Three phage clones, H-phage, I-phage, and L-phage, are of relative high, intermediate, and low affinity for the mouse serum albumin (MSA), respectively. Phage clones were mixed with M− phages at 1:10 ratio, or 10^9^:10^10^ CFU/CFU, and introduced into CMAIL devices. Phages sorted and collected from outlets 1 to 9 were counted separately using conventional phage titer analyses and analyzed using ELISA to confirm antigen interactivity. Only scarce antigen-interactive phages were recovered in the CMAIL device coated with control antigen molecules. In contrast, H-phages were recovered with high efficiency at outlet 1, while I-phages were collected at outlet 6 when target antigens were immobilized to the agarose gel of the device. Only few L-phages were recovered after CMAIL sorting.

We wondered if GMAIL may sort phages into different populations based on their affinity for the antigen. To test, we chose three phage clones having a high (H), intermediate (I), and low (L) affinity for mouse serum albumin (MSA), respectively. The affinity of these phage clones, H-phage, I-phage, and L-phage, was gauged using ELISA, and the absorbance at 450 nm was 2.7, 1.4, and 0.45 for H-phage, I-phage, and L-phage. These phage clones were mixed with M− phages at 1:10 ratio (10^9^:10^10^ CFU/CFU) and loaded into and sorted in separate CMAL devices with their agarose gel coated with either the target antigen, MSA, or the control antigen, VEGF-R2. The BSA blocking step during the preparation of agarose gels was omitted in this set of experiments. As shown in Fig. 4, only scarce antigen-interactive phages were collected when the device was coated with the control antigen molecules, VEGF-R2, suggesting the CMAIL sorting was specific. Positive colony counts were calculated as the product of the colony count (Supplementary Figure 6a) and the ratio of positive ELISA result (Supplementary Figure 6b), which were proportional to the phage concentration and the percentage of antigen-interactive phages, respectively. More phages were recovered when the agarose gel of the CMAIL device was coated with target antigen molecules. Most of the H-phages were concentrated and recovered at outlet 1 with an efficiency of 93%, similar to the efficiency obtained when M+ phages and corresponding target antigens were used in CMAIL. Most of the I-phages were recovered at outlet 6 with an efficiency of 68%. In contrast, when L-phage was evaluated, the phage counts remained low at all the outlets and the total phage count was less than 0.001% to its input count. In summary, phages were sorted and recovered at different outlets of the CMAIL device based on their affinity for the antigen immobilized in the agarose gel. Phages with a strong affinity were highly concentrated at outlet 1, while phages of weaker affinity were collected at outlets further along the direction of the lateral electric field *E*_*L*_, or even largely swept out of the device and not retrieved.

### Five repeated CMAIL operation cycles are possible using the same device

**Figure 5:**
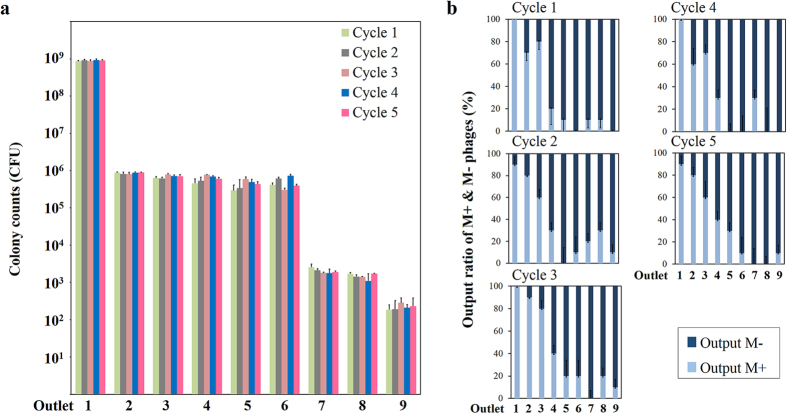
CMAIL devices can be operated for 5 repeated sorting cycles. M+ and M− phages were mixed at a ratio of 10^9^:10^10^ CFU/CFU. Five continuously repeated CMAIL operation cycles, consisting of sample loading, 40-minute sorting, sample harvesting, were conducted using the same devices. (**a**) Similar colony counts of phages collected from 9 individual outlets for 5 repeated CMAIL sorting cycles were obtained. (**b**) ELISA analysis was used to assess the percentage of bacterial colonies infected by M+ phages, and similar results were obtained from samples collected in 5 repeated CMAIL sorting cycles, suggesting the CMAIL device could be operated continuously for at least 200 minutes.

Antigens immobilized in the agarose gel may remain present and viable under the electric field as suggested by the stable fluorescence intensity (Supplementary Figure 1). We wondered if we may reuse a CMAIL device. Phage samples were introduced into the same CMAIL device repeatedly to assess how long we can operate the device continuously without a decline in performance. Antigen-interactive M+ phages and antigen-noninteractive M− phages were mixed at 1:10 ratio (10^9^:10^10^ CFU/CFU). Each cycle consisted of three steps: 1) loading of the phage sample mix into the device, 2) CMAIL sorting for 40 minutes, and 3) collection of samples at each outlet. Five cycles were repeated on the same CMAIL device. Collected phages samples were subjected to conventional phage titer analyses and ELISA. Results, summarized in Fig. 5, indicated that most M+ phages were recovered at outlet 1, and the percentage of M+ phages was above 90% for all the 5 repeated cycles evaluated, suggesting the CMAIL device could be operated continuously for at least 200 minutes.

### CMAIL sorting of a phage library is of favorable recovery rate and purity

**Figure 6:**
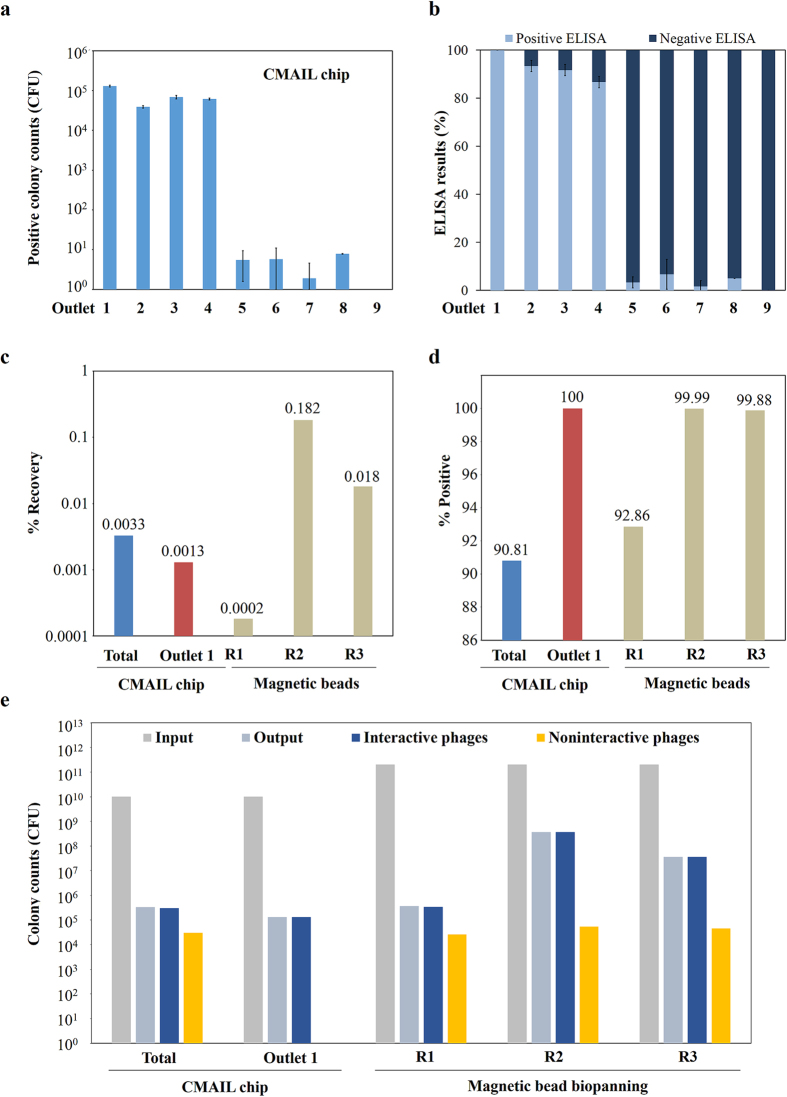
Comparison of CMAIL sorting vs. magnetic bead-based biopanning a phage library. A phage library was screened using both CMAIL chips (*n* = 3) and magnetic beads against the same antigen (VEGF-R2). The interactivity of harvested phages to antigens was assessed using ELISA. (**a**) More than 99.99% of antigen-interactive phages harvested were collected from outlets 1 to 4 of the CMAIL chip. (**b**) More than 94.18% of phages collected from outlets 1 to 4 were antigen-interactive, while phages obtained from outlet 1 were 100% antigen-interactive as gauged by ELISA. (**c**) Percent recovery values were obtained over one round of CMAIL sorting and three rounds of biopanning using magnetic beads. Percent recoveries were determined by the number of harvested phages divided by the number of input phages. Eluted phages were bacterial amplified between rounds of biopanning, and 2.0 × 10^11^ CFU phages were screened for each round of bead-based biopanning. (**d**) Percent positive values were determined by ELISA. The percent positive value was ~100% for phages from the outlet 1 of the CMAIL chip, which was significantly higher than that of the first round of bead biopanning. The percent positive values improved in the second and third rounds of bead-based biopanning. The percent positive values improved in the second and third rounds of bead-based biopanning. (**e**) The summary of phage counts suggested that similar number of antigen-interactive phages were selected using CMAIL chips and magnetic beads, while only ~1/18 input phages were required using CMAIL.

A phage library containing 3 × 10^9^ individual members was screened against the same antigen (VEGF-R2) using both CMAIL and magnetic bead biopanning techniques. 10^10^ CFU phages were introduced into CMAIL chips and sorted for 40 minutes. Phages collected at the outlets were subjected to conventional phage titer analyses and ELISA. As shown in Fig. 6a,b, most phages were collected from outlets 1 to 4 with percent positive values above 85% as gauged using ELISA. The percent positive value was 100% for phages validated from outlet 1. The same phage library was also subjected to three rounds of biopanning using magnetic beads. Eluted phages were bacterial amplified between rounds of biopanning. As shown in Fig. 6c, the percent recovery rates of the initial capture was ~18 times higher using CMAIL than using bead biopanning. The percent recovery rate increased in successive rounds of bead biopanning, indicating the enrichment and amplification of antigen-interactive phages. The percent positive value was ~100% for phages from the outlet 1 of the CMAIL chip, which was significantly higher than that of the first round of bead biopanning (Fig. 6d). The percent positive values improved in the second and third rounds of bead biopanning. The numbers of input and output phages were summarized in Fig. 6e.

## Discussion

Two orthogonal and alternative electric fields, *E*_*V*_ and *E*_*L*_, are utilized to direct phages to navigate through the matrix of agarose fibers functionalized with target antigens in CMAIL. A stronger electric field *E*_*V*_ was used to drive all the phages toward outlets, and the electric field *E*_*L*_ pulled phages laterally and differentially based on their affinity for the antigen. A tug-of-war between the electrophoretic force and the binding force between the antigen and the fusion protein was established on phages in CMAIL. Phages with a strong affinity moved little, while phages with a weaker affinity were pulled laterally under the electric filed *E*_*L*_, resulting in the spatial separation of phages based on their affinity. Both the electric fields and the gel matrix of antigens significantly increased the number of interactions between phages and antigens, and hence the efficiency of CMAIL. In addition, phages are sorted into multiple populations within a single CMAIL cycle and the stringency of sorting can be precisely and conveniently adjusted by modulating the electric fields. In contrast, only two populations of phages, either bound or unbound, are obtained in biopanning. Multiple rounds of biopanning are necessary to sort phages with different stringency, if desired.

M13 phages are filamentous with 6.6 nm in diameter and 880 nm in length^32^. Comparing to other phages that may take shapes from spherical to sophisticated head-tail structure, M13 phages are used most commonly for phage display and are particularly well-suited for CMAIL, as their highly anisotropic shape enables them to pass through pores in the hydrogel with less entrapment. Gel electrophoresis has been employed to measure the mobility of phages and to sort different phage strains using 2% agarose gel at pH 9.5^30^. It has been found that their mobility was largely determined by the length of the phage and the net charge on its surface. In the present study, the length of phages should be similar, since phages expressing fusion proteins contain the same size phagemid. The net charges on the surface of phages are largely determined by the major coat protein pVIII, since there are ~2700 copies per phage, outnumbering the fusion proteins. Therefore, CMAIL sorts phages mainly based on their affinity, not size or surface charge.

Continuous spatial separation. or deterministic lateral displacement, of cells^33^, particles^34^, and DNA molecules^29^ based on their size in an array of micropillars have been demonstrated. The interactions between the object under sorting and the micropillar can be predicted and manipulated. Hence the sorting can be accomplished much faster and with a relatively high degree of precision, compared with sorting in structure-free space or inside the gel matrix. The performance of CMAIL could be further improved by incorporating micro- or nano-pillars in the device, which is being further investigated.

Both NTA and conventional phage titer analyses have been used for assessing the concentration of phages. NTA counts phages directly based on their scattering light or fluorescence images. In contrast, phage titer analysis counts colonies of bacteria infected by phages, which is indirect, lengthy, and more susceptible to errors. However, the sensitivity of both methods is limited by the number of counts per field of view or per culture plate, which is 10^7^ and 10^3^ counts/mL, respectively. Therefore, when phages collected at the outlet of the CMAIL device are below the detection limit, the estimated phage counts may be less reliable. Resistive-pulse sensing, detecting particles upon their passing through a pore, has been investigated to enumeration phages^35^. The method is real-time and label free. However, challenges associated with pore clogging and the agglomeration and coincident passing of phages have to be overcome.

ELISA has been typically used for assessing the outcome of biopanning procedure. However, it only confirms positive binding interactions, since its signal depends not only on the binding affinity, but also the heterogeneity of phage preparation as well as the level and avidity of scFv display. Further evaluation of selected clones involves DNA sequencing, scFv synthesis, as well as competitive ELISA, surface plasmon resonance or other comparable techniques for measuring the binding affinity and kinetics^36^. This approach is costly, time-consuming, and restricts the number of clones that can be evaluated. Several work on the developments of high-throughput assessment of binding affinities has been reported and remained being assessed^37^,^38^.

Biopanning has been implemented using both plate and magnetic bead formats with different performance benchmarks, cost, and time required^39^,^40^,^41^,^42^. Typically magnet beads may provide increased surface area for binding, and thoroughness of washing, and hence better efficiency than plate biopanning^36^,^39^,^43^. Compared with the initial capture efficiency of bead biopanning, the recovery rate of CMAIL is more than an order of magnitude higher. In addition, CMAIL provides a high percentage of antigen-interactive phages immediately after the initial sorting, which can effectively reduce the number of rounds and the chance of repetitive isolation of identical phages augmented from bacterial amplification. Repetitive sorting of identical phages not only waste time and resources, but also may obscure the isolation of low-abundant but valuable phage clones. In conclusion, CMAIL is an automated high-throughput platform capable of sorting phages based on their affinity for the antigen. Compared with the biopanning technique, CMAIL is rapid, cost-effective and of low reagent consumption, and appears to be an approach of choice for affinity sorting of phage-display libraries.

## Methods

### Microfluidic device design and fabrication

The central part of the microfluidic device used in this study was an open 22 mm × 22 mm agarose gel-filled reservoir connected to a 100-μm-wide inlet on one side and nine 1.6-mm-wide outlet channels on the opposite side. The inlet and outlets were all 100 μm deep and made in polydimethylsiloxane (PDMS; Dow Corning, Midland, MI, USA) replicated from a mold fabricated in the photoresist SU-8 (MicroChem, Newton, MA, USA) using a conventional lithography process. The PDMS replica and the glass slide were bonded together after treated with oxygen plasma (18 W, 1% oxygen, 30 s) in a plasma chamber (Harrick Plasma, Ithaca, NY, USA).

### Preparation and modification of agarose hydrogels

Target antigen molecules were immobilized to agarose hydrogels for the selection of interactive phages. In this work, mouse serum albumin (MSA) molecules or vascular endothelial growth factor receptor 2 (VEGF-R2) fragments were linked to agarose via a heterobifunctional photo-crosslinker, sulfosuccinimidyl-6-[4′-azido-2′-nitrophenylamino] hexanoate (sulfo-SANPAH; Merck, Darmstadt, Germany), based on the method described^44^. Briefly, 35 μL sulfo-SANPAH solution at 50 μg/mL in 4-(2-hydroxyethyl)-1-piperazine-ethane sulfonic acid buffer (HEPES; 50 mM, pH 8.5) was mixed with 100 μL 1% (w/v) agarose solution (Sigma Aldrich, St. Louis, MO, USA). The mixture was dispensed onto the open reservoir of the device and exposed to 365 nm UV light for 3 minutes to conjugate sulfo-SANPAH to the agarose. 100 μL solution containing antigen molecules at 8 μg/mL in phosphate buffered saline (PBS; Mediatech, Herndon, VA, USA) was subsequently added to the agarose gel and allowed to react with the NHS-ester group of the sulfo-SANPAH at 4 °C for 24 h. The ligand-modified agarose gel was incubated in 100 μL 3% (w/v) bovine serum albumin (BSA; Sigma Aldrich, St. Louis, MO, USA) solution in PBS at 37 °C for 30 minutes before use.

### Microfluidic sorting of phage clones

Cloned antigen-interactive M+ phages were fluorescently labeled for optical measurements and mixed with antigen-noninteractive M− phages at various ratios before being introduced into the inlet of the CMAIL device. The CMAIL device was soaked in TBE (Tris-borate-EDTA) buffer during operation. Alternating orthogonal electric fields *E*_*L*_ and *E*_*V*_ were generated by using a programmable two-channel DC power supply (Keysight Technologies, Santa Rosa, CA). 15 V and 20 V, corresponding to electric fields of 3.0 V/cm and 7.4 V/cm, were applied alternatively every one minute for 40 minutes to the lateral and vertical pairs of electrodes, respectively. *E*_*V*_ was greater and along the direction from inlet to outlets. Phages at the outlets were collected and subjected to nanoparticle tracking analysis (NTA) or bacterial amplification.

### Nanoparticle tracking analysis (NTA)

Phage samples of concentration ranging from 10^7^ to 10^11^ CFU/mL were introduced into the sample chamber of the NTA instrument (Model LM10 equipped with a 405 nm diode, NanoSight, Amesbury, UK). A 90-second video of scattering microscopy images was captured for each sample to determine the concentration of phages. In experiments when samples contained both M+ and M− phages, M+ phages were fluorescently labelled (Goat anti-Mouse IgG (H + L) secondary antibody; Life technologies, Carlsbad, CA), and a DAPI filter cube was used. Videos of both scattering and fluorescence microscopy images were captured for each sample to determine concentrations of the total and fluorescently-labelled phages, respectively.

### Bacterial amplification

The bacterial strain *E. coli* TG1 was used as the host. Bacteria were grown in 2-YT medium overnight at 30 °C with shaking at 250 r.p.m. until the measured optical density at 600 nm (OD_600_) had reached the value of 0.5. 1 mL of bacterial culture was then incubated with sorted phage (carrying ampicillin resistance) for 30 minutes at 37 °C without shaking. Bacteria were then serial-diluted and inoculated onto 2-YT-AG (2-YT ampicillin-glucose) agar plates. Bacteria were allowed to grow on the plate for 16 h at 37 °C. Bacterial colonies, containing the ampicillin resistance gene from sorted phages, were picked randomly for DNA sequencing or ELISA (enzyme-linked immunosorbent assay).

### DNA sequencing

Bacterial colonies after the bacterial amplification procedure were randomly picked for sequencing. PCR-amplified library inserts from the pIII regions of the phage genome were used for sequencing with the forward primer, 5′-CCA TGA TTA CGC CAA GCT TTG GAG CC-3′; and reverse primer, 5′-CGA TCT AAA GTT TTG TCG TCT TTC C-3′. DNA sequences of the variable regions of the complementary determining regions (CDRs), including CDRH1~3 and CDRL1~3, were extracted, aligned, and translated into amino acid sequences for analysis.

### Enzyme-linked immunosorbent assay (ELISA)

ELISA was carried out to analyze the binding affinity of clonal amplified phages. *E. coli* TG1 that produces pIII phage proteins under the control of the wild-type *lac* promoter was maintained at 37 °C, 250 r.p.m unless otherwise noted during ELISA. Bacterial colonies after the bacterial amplification procedure were randomly picked and grown in 2 YT-AG medium overnight. Each culture was subsequently diluted in 1/16 with 2-YT-A (2-YT ampicillin) medium and re-grown for 2 h. Subsequently, each sample was infected with helper phages (carrying kanamycin resistance) at a final concentration of 4.8 × 10^9 ^CFU/mL in 2YT-A medium for 2 h before kanamycin was added to a final concentration of 25 μg/mL. The bacteria were allowed to grow overnight at 30 °C. 96-well immunoplates for ELISA were coated with 100 μL/well of the solution containing 4 μg/mL antigen molecules (e.g., VEGF-R2 fragments) in PBS for 16 h at 4 °C, and subsequently rinsed with 300 μL/well of PBS for three times. The immunoplates were then incubated with 250 μL/well of blocking buffer containing 5% skim milk in PBS (MPBS) for 2 h at 37 °C, 250 r.p.m before rinsed with 300 μL/well of PBS for three times. The phage suspension diluted in 1% MPBS were centrifuged at 8000 × g, 4 °C for 30 min before 100 μL/well was applied into each well and incubated at 37 °C for 1 h with shaking at 250 r.p.m. The immunoplate was rinsed with 300 μL/well of PBS containing 0.05% Tween-20 (PBST) for three times to remove loosely bound phages. Phage were detected with the monoclonal HRP/anti-M13 antibody solution (100 μL/well, 1:1,000 diluted in 5% MPBS) added to wells and incubated at 37 °C for 1 h. The immunoplate was rinsed with 300 μL/well of PBST three times. TMB, a substrate of HRP, was used for detection and the enzymatic reaction was terminated with 1.0 N sulfuric acid. The ligand-binding activity of phages was quantified by measuring the absorbance at 450 nm with a reference absorbance at 655 nm. The threshold for a positive ELISA result was set to be five times the background intensity.

### Biopanning of phage library

Phages were subjected to both CMAIL and a magnetic bead biopanning procedure. The phage library was prepared as described^36^. Briefly, *E. coli* TG1 cells were transformed with library DNAs by electroporation, and allowed to grow to log phase. TG1 bacteria were infected with Hyperphage and incubated at 37 °C for 30 minutes. Bacteria were then spun down, resuspended in 2-YT medium containing 100 μg/mL ampicillin and 25 μg/mL kanamycin, and grown overnight at 30 °C with shaking at 250 r.p.m. Phages in the supernatant were precipitated with a PEG/NaCl buffer (20% polyethylene glycol 8000, 2.5 M NaCl), and resuspended in PBS. The phage library (10^10^ CFU) were introduced into the inlet of the CMAIL device and sorted for 40 minutes. Phages at the outlets were collected and subjected to bacterial amplification and ELISA. For bead biopanning, the phage library was first pre-incubated with cobalt-coated paramagnetic beads to reduce the number of non-specific binding phages in the library and subjected to three rounds of biopanning. Next, the phage library was incubated with 50 nM histidine-tagged (His-Tag) VEGF-R2 with gentle rotation at 37 °C for 90 minutes. Phages bound with VEGF-R2 were isolated using cobalt coated-magnetic beads that were blocked with 2% MPBS. The beads were washed sequentially with PBST two times, 2% MPBS, PBST two times, 2% MPBS, PBS two times, 2% MPBS, and PBS two times, The bound phages were eluted and bacterial amplified for the next round of biopanning as described above.

### Materials and reagents

The mouse serum albumin (MSA) molecules, vascular endothelial growth factor receptor 2 (VEGF-R2) fragments, M13 bacteriophages, and fluorophore-conjugated anti-M13 antibodies were provided by the Development Center for Biotechnology (New Taipei City, Taiwan)^36^. 2-YT medium containing yeast extract, tryptone and sodium chloride for the cultivation of M13 bacteriophage and Escherichia coli (*E. coli*) were obtained from Amresco (Solon, OH, USA). For conducting ELISA, 96-well immunoplates (Nunc, Rochester, NY, USA), and horseradish peroxidase labeled anti-M13 phage monoclonal antibody (HRP/anti-M13 antibody, GE27-9421-01) were from Sigma Aldrich (St Louis, MO, USA). The substrate for HRP was 3,3′.5,5′ tetramethylbenzidine (TMB) mixed with a stabilized hydrogen peroxide (1-Step Turbo TMB-ELISA, Pierce Biotechnology, Rockford, IL, USA). Cobalt-coated paramagnetic beads were obtained from Invitrogen (Dynabeads His-Tag isolation and pulldown beads, Carlsbad, CA, USA). Hyperphage was obtained from Progen (M13 K07∆pIII, Heidelberg, Germany).

## Additional Information

**How to cite this article**: Hsiao, Y.-H. *et al*. Continuous microfluidic assortment of interactive ligands (CMAIL). *Sci. Rep.* **6**, 32454; doi: 10.1038/srep32454 (2016).

## Supplementary Material

Supplementary Information

## Figures and Tables

**Table 1:** Summary of DNA sequencing results on phages recovered from CMAIL.

	No. of colonies sequenced	No. of colonies sequenced positive for M+ phages	No. of colonies sequenced positive for M− phages	Purity (%)
Outlet 1	10	10	0	100
Outlet 2	9	8	1	89
Outlet 3	9	8	1	89
Outlet 6	9	8	1	89

Phages were retrieved at outlets 1, 2, 3 and 6. Purity was calculated based on the number of colonies sequenced positive for M+ phages divided by the number of colonies successfully sequenced. DNA sequences of M+ phages and M− phages are listed in Supplementary Table 1.
