# Supplementary material for: Continuous microfluidic assortment of interactive ligands (CMAIL)
Source: Sci Rep. 2016 Aug 31;6:32454. doi: 10.1038/srep32454 (PMC5006012; doi:10.1038/srep32454)

**Continuous microfluidic assortment of interactive ligands (CMAIL)**

Yi-Hsing Hsiao^1,2^, Chao-Yang Huang^3^, Chih-Yung Hu^3^, Yen-Yu Wu^3^, Chung-Hsiun Wu^3^, Chia-Hsien Hsu^1,2^ and Chihchen Chen^1,4,*^

**Supplementary Figures and Tables**


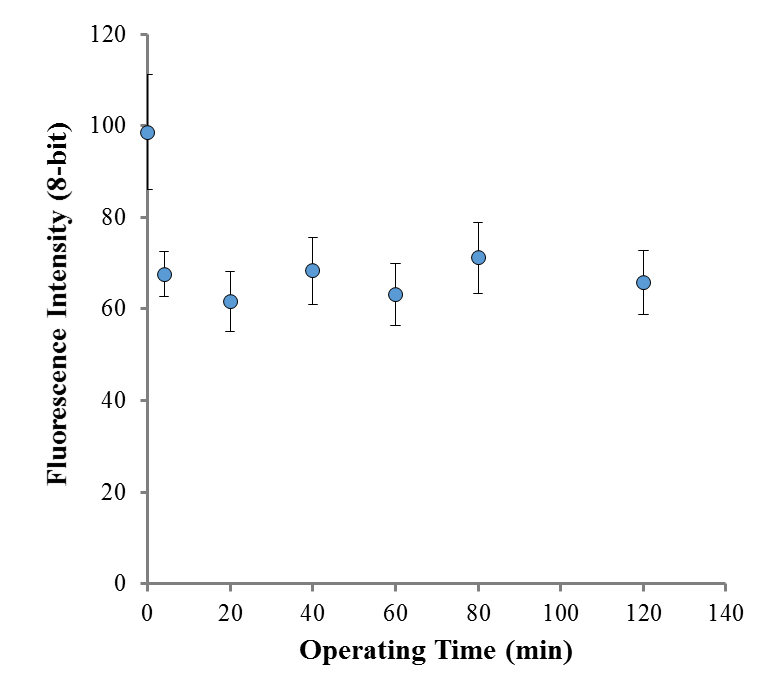


**Supplementary Figure 1. Immobilization of antigen molecules to the agarose gel was stable for at least 120 minutes under the electric field.** Antigen molecules were conjugated to the agarose gel via sulfosuccinimidyl-6-[4’-azido-2’-nitrophenylamino] hexanoate (sulfo-SANPAH) crosslinkers. The gel was subsequently incubated with solution containing fluorescently labeled antibody against the antigen at room temperature for 4 h before an electric field of 7.4 V/cm was applied across the gel. Fluorescent intensity measurements suggest antigen molecules remain present in the agarose gel for the duration assessed. The initial drop in fluorescence intensity may be due to the removal of unbound antibodies by the electric field (*n* = 8).


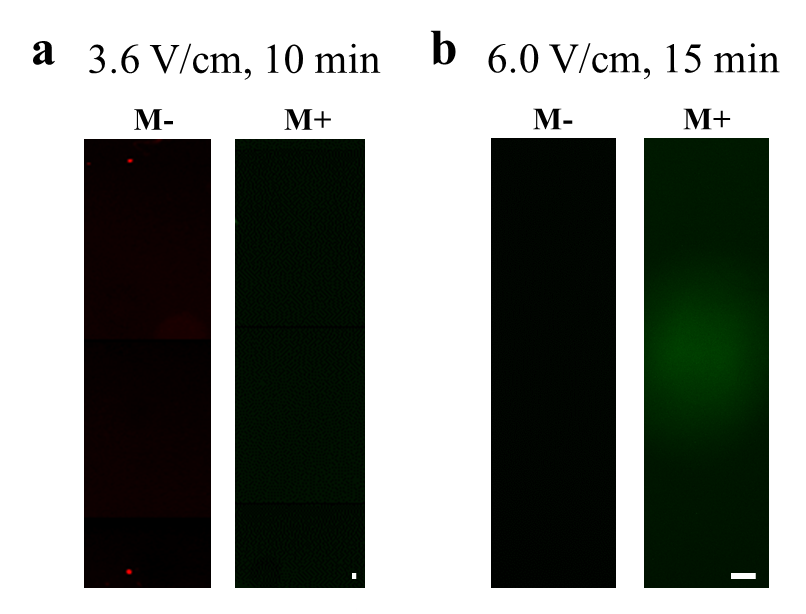
**Supplementary Figure 2. Fluorescence micrographs of migrating phages in antigen-immobilized 1% agarose gels.** Antigen-interactive cloned phages, or M+ phages, were labeled green, while non-interactive cloned phages, or M- phages, were labeled red. (**a**) M- phages were visible in the gel. Images were taken after the application of an electric field of 3.6 V/cm to the gel for 10 minutes. (**b**) M+ phages migrated toward the anode under the electric field of 6.0 V/cm. Images were taken 15 minutes after the start of electrophoresis (scale bar = 100 μm).


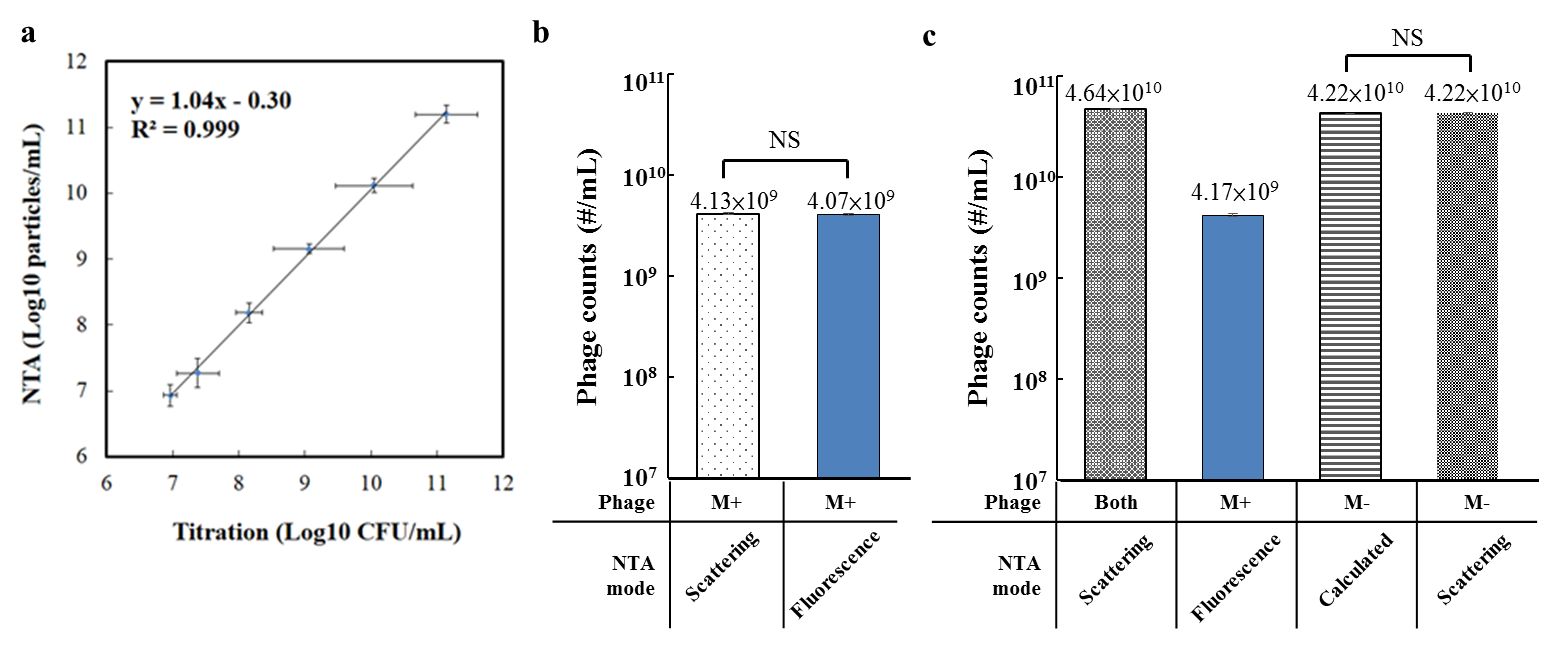
**Supplementary Figure 3. Rapid enumeration of phages and fluorescently-labeled phages using nanoparticle tracking analysis (NTA).** (**a**) Phage counts obtained by using NTA and conventional phage titer analyses are comparable for phage concentrations greater than 10^7^ particles/mL (*n* = 3). (**b**) NTA counts of fluorescently labeled M+ phages obtained under the scattering mode and fluorescence mode are comparable (*p*-value = 0.48, Student’s *t*-test, *n* = 3); NS: not-significant. (**c**) M+ and M- phage sample were mixed and subjected to NTA. Total phage counts were obtained under the scattering mode, while M+ phage counts were obtained under the fluorescence mode. M- phage counts calculated using the difference of the total counts and M+ phage counts were comparable to the concentration of M- phages measured before mixing (*p*-value = 0.94, Student’s *t*-test, *n* = 3); NS: not-significant.


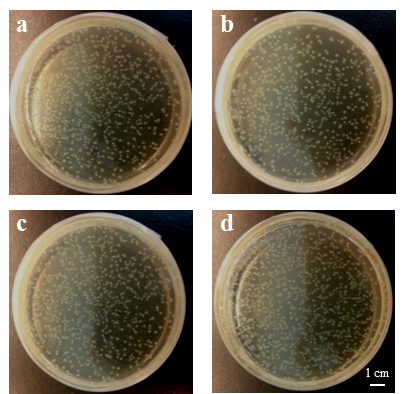
**Supplementary Figure 4. Images of phage-infected TG1 bacterial colonies.** Bacteria were titered and grown overnight on 2-YT ampicillin-glucose (2-YT-AG) agar plates after the infection by phages sorted using CMAIL and recovered at (**a**) outlet 1, (**b**) outlet 2, (**c**) outlet 3, and (**d**) outlet 6.


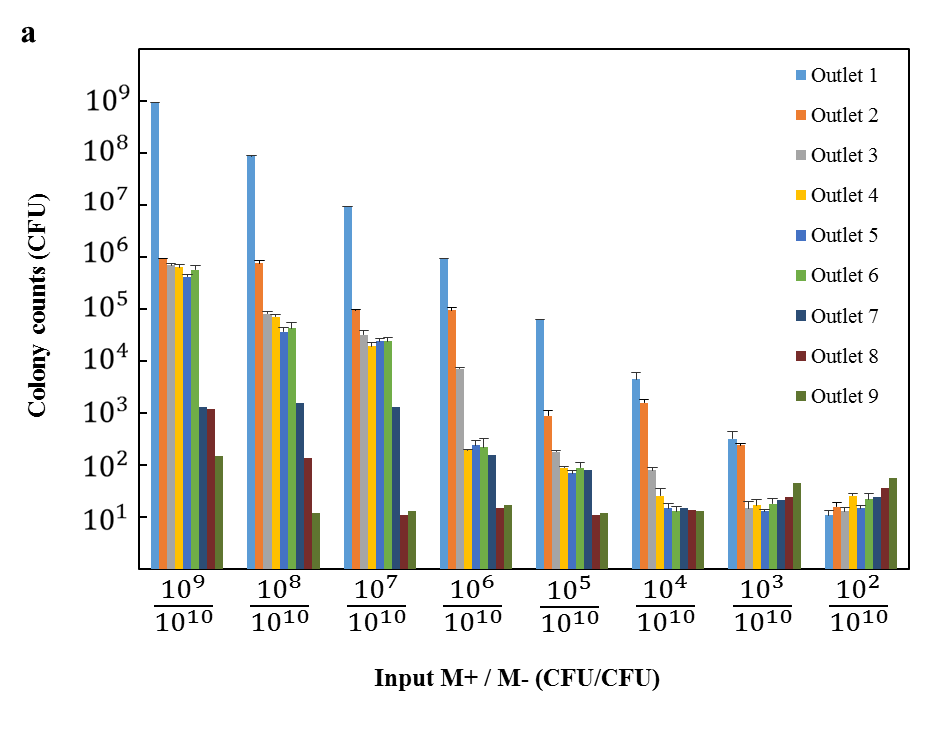

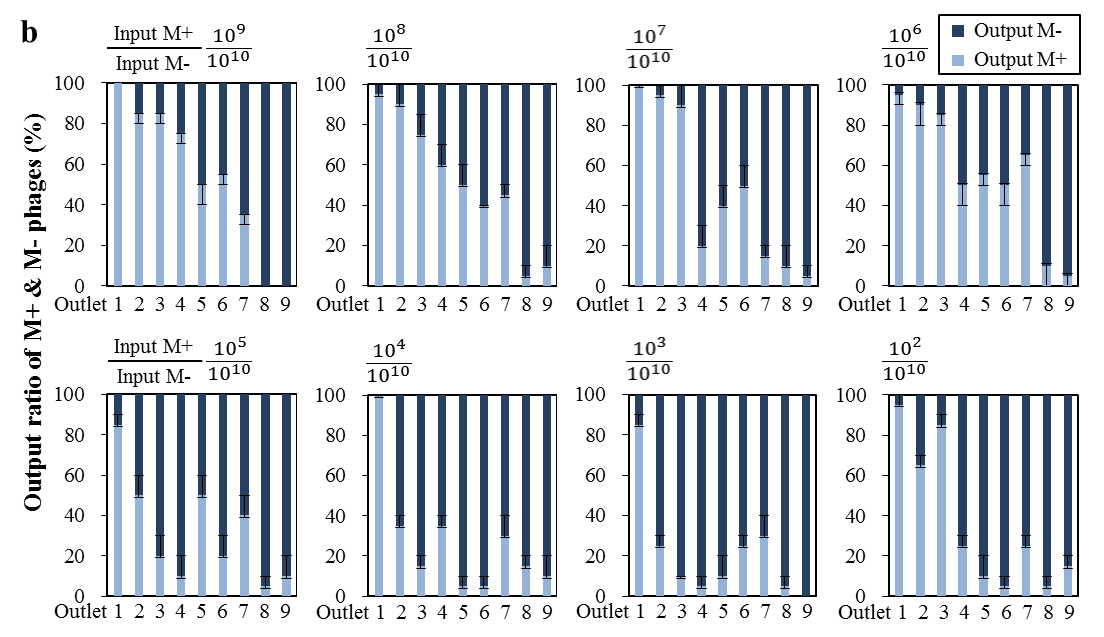


**Supplementary Figure 5. Phage counts and ELISA analysis on cloned antigen interactive (M+) and noninteractive (M-) phages mixed at different ratios after CMAIL.** The input sample contained M+ phages ranging from 10^2^ to 10^9^ CFU and 10^10^ CFU M- phages. (**a**) Phages collected at outlets 1 to 9 of a CMAIL device were counted separately using conventional phage titer analyses. (**b**) ELISA analysis was used to assess the percentage of bacterial colony infected by M+ phages from samples of various mixing ratios collected at the outlets.


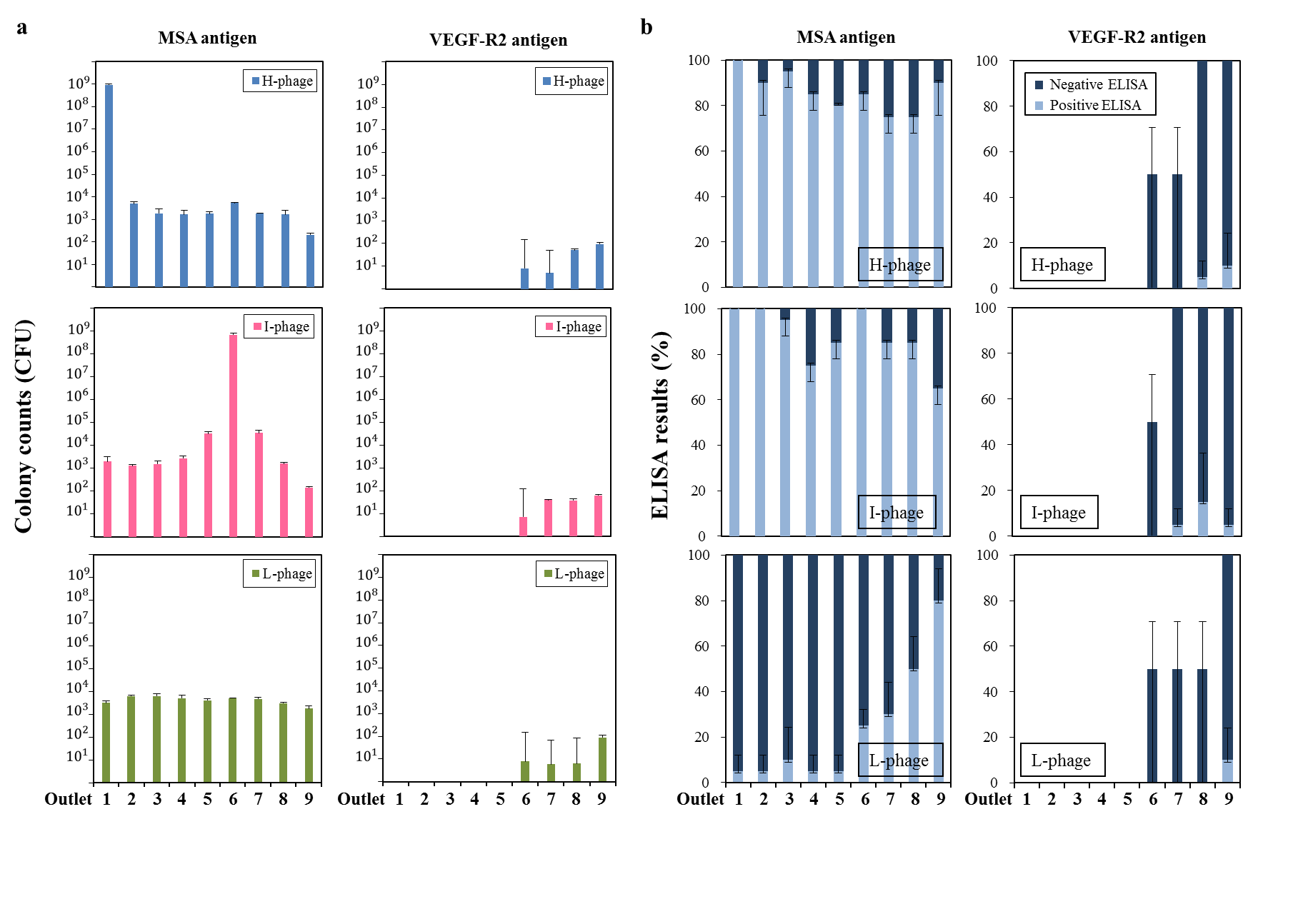


**Supplementary Figure 6. Bacterial colony counts and ELISA analysis of phage clones after sorting using CMAIL devices coated with either target or control antigens.** (**a**) Three phage clones, H-phage, I-phage, and L-phage, are of relative high, intermediate, and low affinity for mouse serum albumin (MSA), respectively. Phage clones were mixed with M- phages at 1:10 ratio, or 10^9^:10^10^ CFU/CFU, and introduced into CMAIL devices. Phages sorted and collected from outlets 1 to 9 were counted separately using conventional phage titer analyses. More phages were recovered when the agarose gel of the CMAIL device was coated with target antigen molecules, MSA, and very few phages were collected when the control antigen molecules, VEGF-R2, were used. The outlet number of the peak phage counts were outlet 1 and outlet 6 when H-phages or I-phages were used, respectively. There was no obvious peak when L-phage was evaluated. (**b**) ELISA analysis was performed to assess the percentage of randomly-picked bacterial colonies producing antigen-interactive phages. Phages amplified from most colonies were measured positive when H-phages or I-phages were used. In contrast, the percentage was low when the control antigen molecules were used to coat the agarose gel, suggesting the sorting was specific.

**Supplementary Table 1. DNA sequences of variable domains of scFv fusions in 40 single colonies infected by phages sorted and collected at different outlets of the CMAIL device.** Amino acid sequences of CDRH1, CDRH2, and CDRH3 domains of heavy chains and CDRL1, CDRL2, and CDRL3 domains of light chains in the scFv fusions of randomly picked phage clones are shown. Sequences identified for positive phages are boxed, and sequences identified for negative phages are in red font.


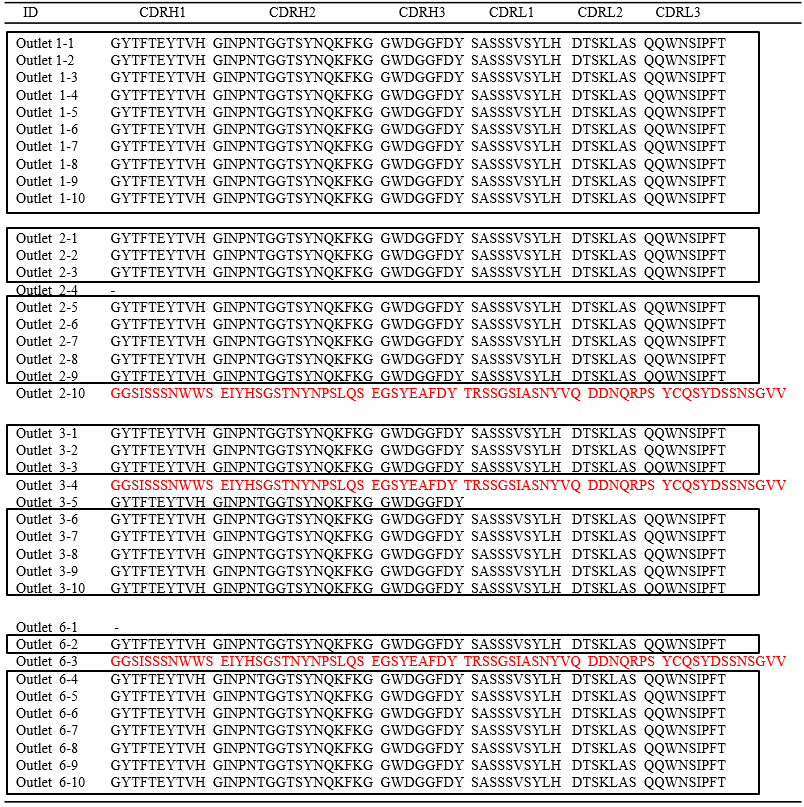

Supplement: Supplementary Information [file srep32454-s1.doc]
